# Molecular Docking and Molecular Dynamics (MD) Simulation of Human Anti-Complement Factor H (CFH) Antibody Ab42 and CFH Polypeptide

**DOI:** 10.3390/ijms20102568

**Published:** 2019-05-25

**Authors:** Bing Yang, Shu-Jian Lin, Jia-Yi Ren, Tong Liu, Yue-Ming Wang, Cheng-Ming Li, Wen-Wen Xu, You-Wen He, Wei-Hong Zheng, Jian Zhao, Xiao-Hui Yuan, Hua-Xin Liao

**Affiliations:** 1 Institute of Biomedicine, Jinan University, Guangzhou 510632, China; candyYang_3940@163.com (B.Y.); linshujian18@163.com (S.-J.L.); liutong_smile@163.com (T.L.); ws_wym@126.com (Y.-M.W.); cli816@163.com (C.-M.L.); xuwenwen@jnu.edu.cn (W.-W.X.); 2 Guangdong Provincial Key Laboratory of Bioengineering Medicine, Guangzhou 510632, China; 3 Zhuhai College of Jilin University, Zhuhai 519041, China; jiayi80@126.com; 4 National Engineering Research Center of Genetic Medicine, Guangzhou 510632, China; 5 Department of Immunology, Duke University Medical Center, Durham, NC 27503, USA; youwen.he@duke.edu; 6 Zhuhai Trinomab Biotechnology Co., Ltd., Zhuhai 519040, China; zwh13901154411@139.com (W.-H.Z.); niezi581@163.com (J.Z.)

**Keywords:** complement factor H (CFH), molecular docking, molecular dynamics (MD) simulation, computational alanine scanning (CAS), experimental alanine scanning (EAS), complementarity-determining region (CDR)

## Abstract

An understanding of the interaction between the antibody and its targeted antigen and knowing of the epitopes are critical for the development of monoclonal antibody drugs. Complement factor H (CFH) is implied to play a role in tumor growth and metastasis. An autoantibody to CHF is associated with anti-tumor cell activity. The interaction of a human monoclonal antibody Ab42 that was isolated from a cancer patient with CFH polypeptide (pCFH) antigen was analyzed by molecular docking, molecular dynamics (MD) simulation, free energy calculation, and computational alanine scanning (CAS). Experimental alanine scanning (EAS) was then carried out to verify the results of the theoretical calculation. Our results demonstrated that the Ab42 antibody interacts with pCFH by hydrogen bonds through the Tyr315, Ser100, Gly33, and Tyr53 residues on the complementarity-determining regions (CDRs), respectively, with the amino acid residues of Pro441, Ile442, Asp443, Asn444, Ile447, and Thr448 on the pCFH antigen. In conclusion, this study has explored the mechanism of interaction between Ab42 antibody and its targeted antigen by both theoretical and experimental analysis. Our results have important theoretical significance for the design and development of relevant antibody drugs.

## 1. Introduction

Complement factor H (CFH) is a major soluble glycoprotein that is expressed by normal epidermal cells with a molecular weight of 155 kDa [1,2]. The main function of CFH is to protect the host cells from complement system-mediated attack and destruction through inhibiting the alternative complement activation pathway, i.e., preventing the deposition of complement component 3-b (C3b) on the surface of target cells [3,4]. Certain malignant tumor cells escape the body’s tumor clearance through over-expressing CFH, which is considered to be one of the important mechanisms by which malignant tumors escape from the host immunity [3,5].

In some patients with non-small cell lung cancer, serum is found to contain autoantibodies that specifically recognize a peptide epitope PIDNGDIT that was located on the CFH SCR19 domain [4,6], which is an important region for its binding to the membrane of C3b-expressing cells [2,5]. A previous report also showed that the anti-pCFH antibodies recognize and bind CFH on the surface of tumor cells, and thereby facilitate the activation of complement system, which in turn damages the tumor cells by both complement-dependent cytotoxicity (CDC) and antibody-dependent complement-mediated cytotoxicity (ADCC), and it ultimately suppresses tumor growth and prevents tumor metastasis [6,7].

Ab42 is a human-derived high-affinity anti-pCFH monoclonal antibody (under Chinese invention patent application number: 201611201316.2). Ab42 was isolated in our laboratory from the peripheral blood mononuclear cells (PBMC) of a malignant glioma patient while using single memory B lymphocyte sorting and the reverse transcription-polymerase chain reaction (RT-PCR) method [8]. As autoantibody to CHF is associated with anti-tumor cell activity [8], anti-pCFH Ab42 as a native human antibody has potential for the development of anti-tumor drugs.

Understanding how an antibody interacts with its targets is critical for the development of antibody as therapeutic drug. Molecular docking [9,10] and molecular dynamic (MD) simulation [11,12] methods provide an advantageous means for studying the interaction between antigen and antibody. Amber99sb originally developed from the amber99 force field has improved the parameters for the prediction of protein secondary structure [13,14,15] and it is especially suitable for MD simulation of proteins. The results are often consistent with the experimental observations. Our previous studies have screened different solvent water models and force field calculations in the MD simulation system, and proved that amber99sb is the best candidate model for studying antigen-antibody interactions [16].

Therefore, the present work has applied the amber99sb model to study the interaction between Ab42 antibody and pCFH by molecular docking, MD simulation, and CAS [17,18], and it has successfully identified the binding epitopes of Ab42. Finally, the results of theoretical calculations were further verified by both experimental alanine scanning (EAS) [1,19] and enzyme-linked immunosorbent assay (ELISA).

## 2. Results and Discussion

### 2.1. The Three-Dimensional (3D) Structure of Ab42

**Figure 1:**
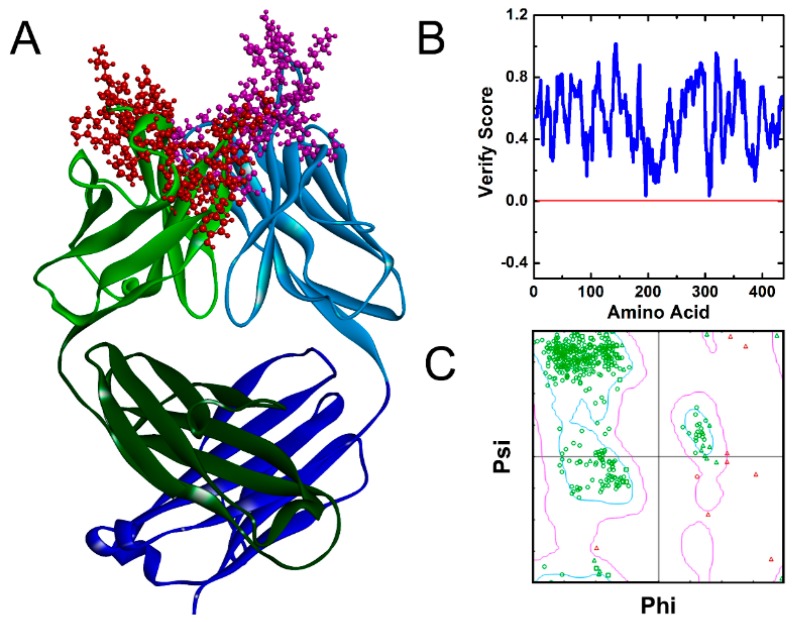
The theoretical model and the structure evaluation of Ab42. (**A**) Ab42 Fab theoretical model with the heavy chain complementarity-determining regions (CDRs) displayed by red color and light chain CDRs displayed by purple spheres respectively; (**B**) Profile_3D verification result of the Ab42 model with residues exhibiting reasonable folding; and, (**C**) the Ramachandran plot analysis shows phi-psi torsion angles of all residues in the structure, and Glycine residues are separately identified by triangles.

Only the sequence of Fab fragment of Ab42 antibody was included in the study to more accurately explore the interaction between the antigen and the antibody and to minimize the calculation (Figure 1A). We used the Verify Protein (Profiles-three-dimensional (3D)) program under the Homology Modeling module to examine the compatibility scores of each amino acid residue in its primary sequence on both the homology-modeled structure and the corresponding 3D spatial structure.

Figure 1B shows that the Verify Score of Ab42 Fab antibody structure, all the amino acid scores are greater than zero, which indicates that the amino acid sequence of Ab42 antibody is compatible with its corresponding 3D structure. The stereo-chemical accuracy of the model, including the rationality of structural parameters, such as bond length, bond angle, and dihedral angle, was evaluated by the Ramachandran plot analysis method. The analysis shows that most of the amino acid residues (except Glycine) are located in the “core” regions that represent the most favorable combinations of phi-psi values, which suggests that the bond lengths, bond angles, and dihedral angles of the entire molecule are reasonable (Figure 1C). Glycine residues are separately identified by triangles, as these are not restricted to the regions of the plot appropriate to the other side-chain types. These results are similar to the template for homology modeling and they are also consistent with the Profiles-3D predictions, suggesting that the structure of Ab42 antibody was precisely optimized by energy minimization with correct stereo-structure accuracy.

### 2.2. Molecular Docking

**Figure 2:**
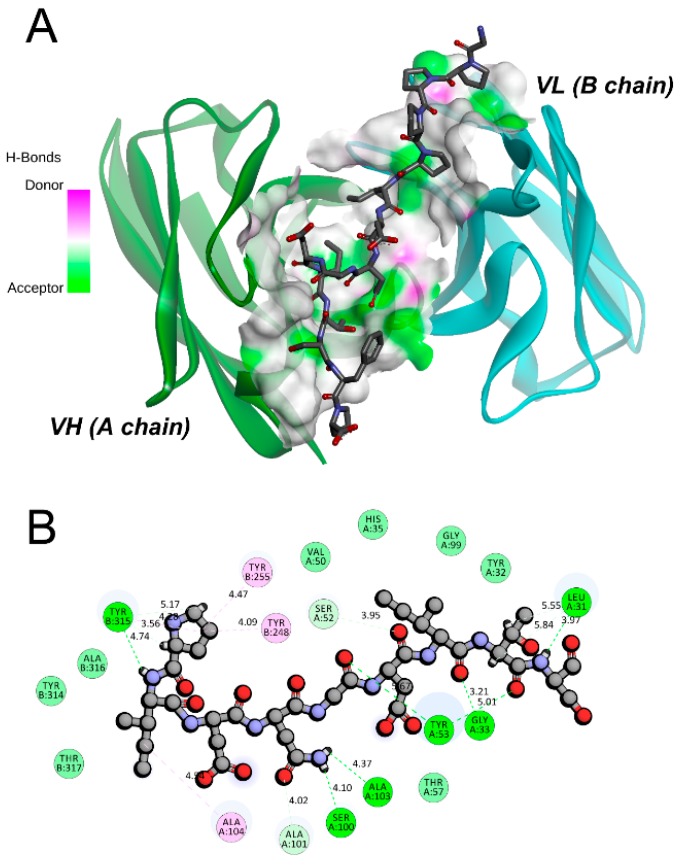
The interaction of Ab 42 with pCFH. (**A**) atomic surface contact of pCFH with Ab42 CDR; (**B**) two dimensional diagram of the interaction between pCFH and Ab42, with pCFH displayed by ball and stick and the key residues in CDR displayed by disc presentation.

The ZDOCK program was used to perform the molecular docking between the pCFH and Ab42 proteins to achieve the highest docking pose (Figure 2). Our results showed that the entire pCFH molecule spans around the CDR of Ab42 molecule (Figure 2A), and subsequent analysis further revealed that pCFH strongly interacts with the surface amino acid residues of Ab42, mainly by hydrogen bonding and hydrophobic interaction (Figure 2B), both of which play major roles in the binding of pCFH to the Ab42 antibody. As shown in Figure 2B, pCFH forms hydrogen bonds with the sites of Tyr315, Ala103, Ser100, Gly33, Ser52, and Leu31 on the CDR of Ab42 antibody, and it also hydrophobically interacts with the sites of Tyr248, Tyr255, and Ala104.

In the docking model, 15 intermolecular hydrogen bonds were formed in the Ab42-pCFH complex (Table 1), with an average length of approximately 2.0 angstroms, indicating that they play the most important roles in the binding between Ab42 and pCFH during the complex formation, followed by the hydrophobic interactions.

### 2.3. Stability Analysis of Ab42-pCFH Complex

The MD simulation can be used to solve the energy barrier problem that EM calculation cannot overcome, and the trajectory of MD in equilibrium can be used for the calculation of binding free energy using the molecular mechanics/Poisson-Boltzmann solvent accessible surface area (MM-PBSA) method. In this study, the Ab42-pCFH complex was subjected to a single 600 ns MD simulation under the force field of Amber99sb-spce for analysis. The total energy and the root mean square deviations (RMSDs) were subsequently analyzed to evaluate the balance of trajectory.

**Figure 3:**
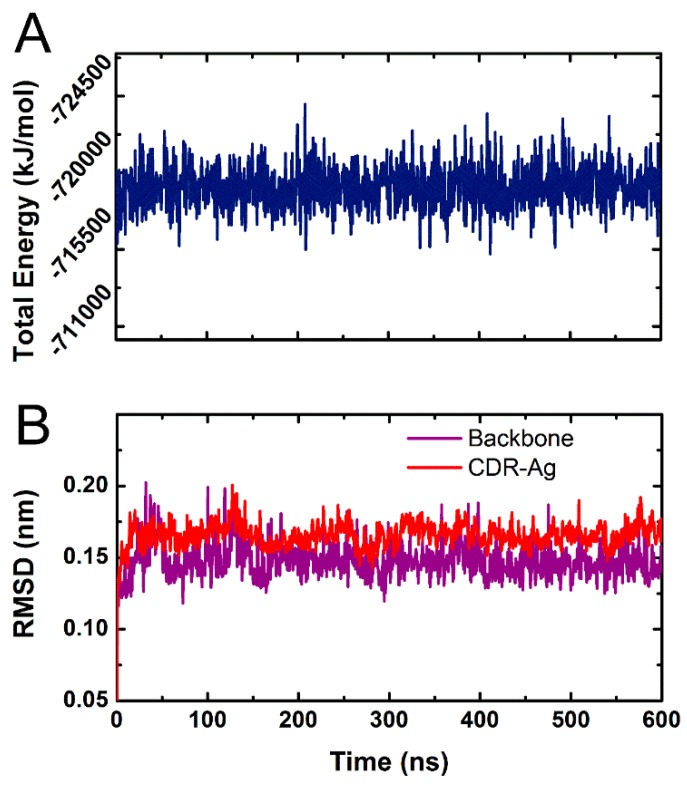
The total energy and root mean square deviations (RMSD) as functions of the Ab42-pCFH molecular dynamic (MD) simulation time. (**A**) the total energy of whole Ab42-pCFH structure; (**B**) the backbone RMSD of whole Ab42-pCFH structure labeled with puple color and the CDR-Ag RMSD of whole Ab42-pCFH structure labeled with red color.

As shown in Figure 3A, the system reached to equilibrium at approximately 50 ns of the analysis. We used the index tool of Gromacs (Make_ndx) to define the CDRs of Ab42 and the pCFH as a molecular group, because the interaction of antigen and antibody is mainly between the epitope of the antigen and the CDRs of the antibody, in the RMSD analysis (CDR-Ag group). In the 600 ns MD process, in addition, to analyze the RMSD of the total backbone, the variation curve of CDR-Ag was also analyzed (Figure 3B). The results of RMSD analysis of the CDR-Ag group showed that the CDRs of the antibody stably interacted with the pCFH, as evidenced by the observation in which the RMSD values remained within the range of 0.1 nm, indicating that the CDR regions of the antibody maintained a stable binding state with pCFH.

### 2.4. MM-PBSA and Energy Decomposition

**Figure 4:**
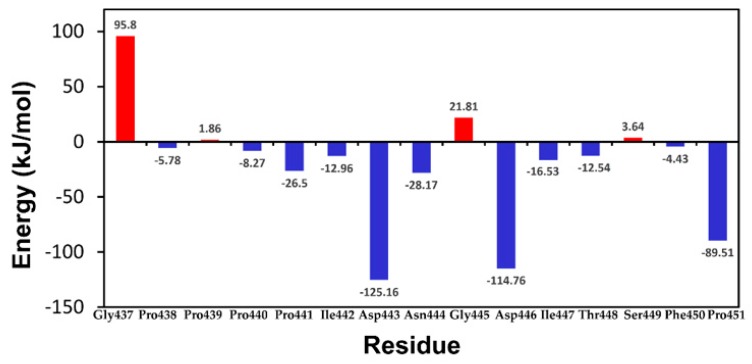
Amino acid energy decomposition of pCFH. The red bar or the blue column indicates the binding energies positive or negative respectively after the substitution. The smaller the Amino acid energy decomposition value (kJ/mol), the greater the contribution of this amino acid to the binding of antigen to antibody.

The equilibrium phase of the three kinetic simulations of the Ab42-pCFH complex structure was selected in order to further explore the interaction mechanism between pCFH and antibody Ab42. Binding free energy and residue decomposition were then calculated by using MM-PBSA. The final relative binding free energy of Ab42-pCFH complex is −617.381 ± 50.617 KJ/mol, which is a typical binding free energy between antigen and antibody. We further analyzed the energy contributions of pCFH and the key amino acid sites on the CDRs of Ab42. Figure 4 showed that almost all of the amino acids (PRO441, ILE442, ASP-443, Asn-444, ASP-446, ILE-447, THR448, and PRO451) in pCFH sequence, except for GLY437, Pro439, GLY445, and Ser449, which did not interact with the antibody displayed negative energy values, strongly suggesting that these residues on the polypeptide are involved in the binding of pCFH to Ab42 antibody. From the perspective of energy decomposition, Pro441, Asp443, Asp444, Asp446, and Pro451 contribute more to the energy of the bond. 

### 2.5. Comparison of CAS and EAS

Alanine substitution of an amino acid replaces its reactive group with a small neutral methyl group on the side chain, exerting little effect on the whole protein structure, and is hence commonly used in observing the functional impact of amino acids on proteins. Therefore, this study used EAS to further validate the results that were obtained by using CAS.

The free energy changes after amino acid substitution with alanine residue were mainly evaluated by using CAS. An energy value of >0.5 kcal/mol after the mutation indicates that the structure is unstable and that particular amino acid residue(s) may play an important role in stabilizing the structure, whereas a post-mutational energy value of <−0.5 kcal/mol means that the structure remains stable and that the original amino acid in situ is not conducive to the structural stability. Similarly, a substitution energy change between 0.5 and −0.5 kcal/mol indicates that there is no significant effect of the amino acid on the structural stability before and after the mutation, i.e., these amino acids play little roles on the structural stability.

**Figure 5:**
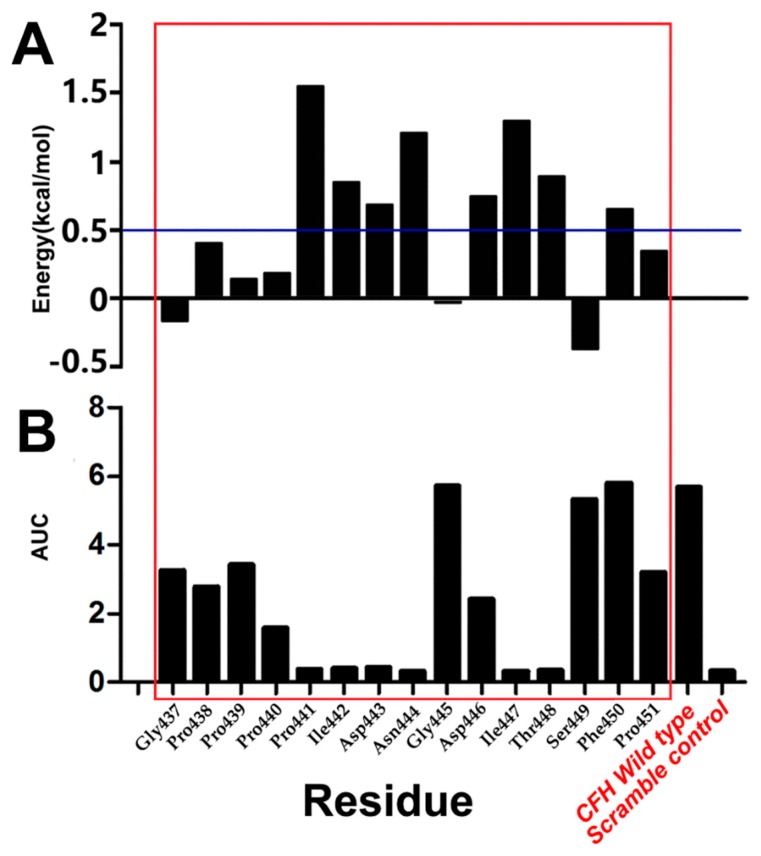
Results of computational alanine scanning (CAS) and experimental alanine scanning (EAS). (**A**) CAS, Pro441, Ile442, Asp443, Asn444, Asp446, Ile447, Thr448, and Phe450 had an energy of >0.5 kcal/mol after mutation to Ala; (**B**) EAS, the area under the curve (AUC) of the Pro441, Ile442, Asp443, Asn444, Ile447, and Thr448 is almost as with the negative control of Scramble control.

CAS data showed that amino acid substitution with Ala at each of the sites at Pro441, Ile442, Asp443, Asn444, Asp446, Ile447, Thr448, and Phe450 resulted in the energy of >0.5 kcal/mol (Figure 5A), which suggests that the amino acids at these sites play a key role in stabilizing the Ab42-pCFH complex and a change of amino acids at these positions may impact the conformational changes of the entire molecule.

As shown in Figure 5B, the area under the curve (AUC) was measured and plotted for the binding of serially diluted antibody Ab42 with the wild-type peptide and mutant peptides with Ala substitution in the ELISA assays. The results represent a pharmacokinetic parameter for the bioactivity of the drug (the extent to which the drug is absorbed and utilized in the human body). The larger the AUC, the higher the bioactivity, and vice versa. As shown in Figure 5, the binding, reflected as AUG, of Ab42 to mutant peptides with amino acid residue substitution at the Pro440 and Asp446 was approximately 30% and 50% of the binding to the wild type CFH peptide, respectively. The binding of Ab42 to CFH peptides with the substitution of the amino acid residue at Pro441, Ile442, Asp443, Asn444, Ile447, and Thr448 was less than 10% of the binding to the wild type CFH peptide, which was almost at the same level of binding to the scrambled peptide negative control (a random sequence with same composition of amino acids as the wild-type CHF peptide). These results indicate that the amino acids at these sites are important residues. It was found that the importance of certain amino acids in antigen-antibody identified by EAS and CAS were not always consistent. In EAS, a biotin tag was added to each of the synthesized CFH peptides to minimize or avoid the block of antigenic epitopes due to coating peptide directly to the ELISA micro-titer plates, which was captured by streptavidin on ELISA plates to project CFH peptides for better exposure of antigenic epitope. However, in CAS, the importance of amino acids is reflected by the energy change of the mutants, and the energy change did not directly correspond to the OD value that was obtained in the ELISA experiment. Such inconsistency of the results that were obtained by EAS and CAS might be due to the fact that the presence of a biotin tag might sometimes have an adverse effect on the antigen-antibody binding.

We have also noticed that the results of alanine scanning were not completely consistent with the results of residue decomposition that were calculated by MM-PBSA, especially for the ASP446. The contribution of an amino acid to the binding free energy reflects its importance in the complex structure during the alanine scanning process in both CAS and EAS. When a given amino acid residue was mutated, it not only had an effect on the original site in the structurally complex, but it might also influence the structure of the amino acids around it or even on the amino acids distanced away from the site. In general, our data indicated that the six amino acids PRO441, ILE442, ASP-443, Asn444, Ile447, and Thr448 are the key amino acids for the formation of a stable complex.

### 2.6. Distance Monitoring of Key Amino Acids

**Figure 6:**
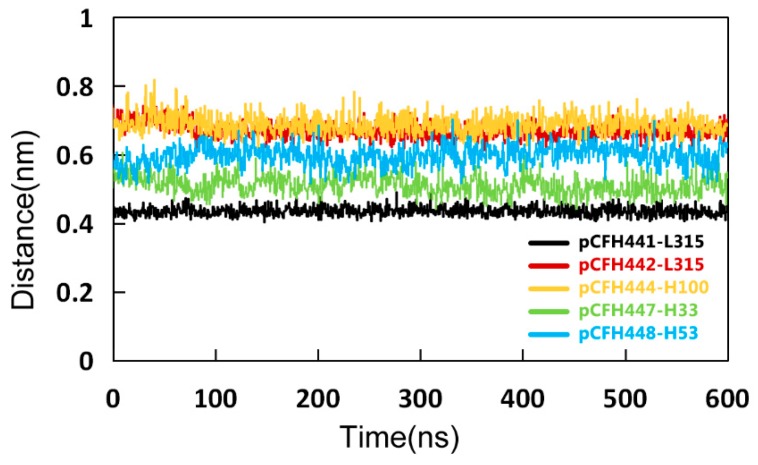
The Key amino acid distances between pCFH and Ab42. The distances of pCFH441-L315, pCFH442-L315, pCFH444-H100, pCFH447-H33, and pCFH448-H53 were represented by black, red, yellow, green, and blue curves, respectively.

The change in distance over time was calculated between several pairs of amino acids through which pCFH interacts with Ab42 by hydrogen bond formation in order to find out the specific bonds between the key amino acids in the structure of Ab42-pCFH complex. As shown in Table 1, amino acids on Ab42 interacting with pCFH are located in the CDRs of the antibody, while the key amino acids on the polypeptide are Pro441, Ile442, Asn444, Ile447, and Thr448, which form hydrogen bonds, respectively, with the residues Tyr315, Ser100, Gly33, and Tyr53 on the CDRs of Ab42. The relevant distance for pCFH is also very stable (Figure 6), implying the stability of the CDR structure. As shown in Figure 4, the decomposition energy of these amino acids in the CDRs are also negative, suggesting that the amino acids at these sites significantly contribute to the antigen-antibody binding.

## 3. Materials and Methods

### 3.1. Experimental Preparation

The structure of CFH polypeptide was retrieved from the PDB database (PDB_ID: 5EA0) [1]. After initial structural processing, the Dmol3 module in the Discovery Studio v4.5 (DS45) software [20] was used for the quantum chemical optimization. Subsequently, a density functional theory method of B3LYP [12] was used to refine the conformation of CFH polypeptide. The maximum SCF cycle was set to 300. The calculated non-bond interaction value was set to 1.4 nm.

Native human monoclonal antibody Ab42 was isolated from sorted single human B cells using protocol, as described [8]. Briefly, single antigen-specific human B cells were sorted by flow cytometry from the peripheral blood mononuclear cells (PBMC) of a malignant glioma patient while using fluorescence dye-labeled CFH peptide (GPPPPIDNGDITSFP). The variable gene segments of immunoglobin (Ig) heavy-(VH) and light-chain (VL) were isolated from the sorted single B cells by RT/PCR. The isolated Ig VH and VL gene segments were sequenced and used for assembling expression constructs for the production of recombinant antibody [8]. The specific binding of the isolated antibody Ab42 was tested and confirmed for binding to CFH polypeptide in ELISA. Recombinant Ab42 Fab antibody was produced while using the protocol, as described previously [8,21] and it was confirmed to retain the same ability to bind to the antigen as the intact full-length IgG1 antibody. Therefore, the Fab fragment of the Ab42 antibody was used in the process of homology modeling to improve the calculation performance and minimize the calculation.

The Ab42 Fab fragment antibody has 217 amino acids in the heavy chain and 219 amino acids in the light chain. Based on the IMGT numbering system (http://www.imgt.org/IMGT) complementarity-determining regions (CDRs) are part of the variable region of immunoglobulin heavy (H)-and light-chain (L) and they play critical role in binding to antigen. Table 2 summarizes the amino acid sequences and position of HCDR1, HCDR2, HCDR3, as well as L-CDR1, L-CDR2, and L-CDR3 of Ab42.

The 3D structure of Ab42 was established by using the homology modeling method, as described [20,22]. Blast search was performed to identify the best candidate templates for homology modeling in the PDB database (http://www.rcsb.org). PDB_ID:4G5Z_H (Identity: 83%, resolution 1.83 angstroms) and 3O2D_L (Identity: 89% resolution 2.19 angstroms) were identified to be the templates with the closest similarity for the heavy chain and light chain of Ab42. The best template 1BGX (resolution 2.3 angstroms) for the interface was also identified. The initial structure of the Ab42 Fab antibody was then modeled with DS_Model Antibodies module in the DS45 package. Next, the structure of both HCDRs and LCDRs were optimized using the Model Antibody Loops program of DS45. A total of 1000 output theoretical models were generated and sorted by the score of PDF Total Energy, which is the sum of the scoring function value as well as the DOPE score based on statistical potential as a measure of the model quality. The best theoretical model was selected by using the PDF total energy and DOPE score (the lower the score, the better the model). Finally, the charmm27 force field was used in the Generalized Born with a simple Switching (GBSW) solvent model. The energy minimization (EM) was performed on the DS_Charmm module. The output structure with convergence value lower than 0.4184 kJ/(mol·nm) was the initial structure for the molecular docking calculation.

### 3.2. Molecular Docking

The molecular docking as a calculation method was used to predict the binding mode of receptors to ligands. The docking calculation of pCFH with Ab42 was performed by the ZDock [23,24], utilizing a fast Fourier transform (FFT) algorithm [25] to search the spatial degree of freedom between receptor and ligand, and to report each of the binding modes with an energy scoring function under the DS45 software. The pCFH and Ab42 molecules were assigned to the all-atom Charmm27 force field [26]. The resulting models were then minimized by using the steepest descent (SD) method and the conjugate gradient (CG) method until the whole system convergence criterion reaching to 0.4184 kJ/(mol·nm). pCFH was the ligand and Ab42 was the receptor whose CDRs was defined as the binding region in the docking calculation. The RMSD Cutoff and the Interface Cutoff were set to six and nine angstroms, respectively [27,28]. A total of 2000 docking poses and 100 cluster were generated.

In filtering process for these poses, the angular step size was set to six degrees and the distance Cutoff was set to 10 angstroms as described [29]. All of the docking poses obtained were scored by ZRank [30]. The best energetic pose conformation of the largest cluster of complexes was selected for analysis by the RDock [31] program. The simulated annealing method was used to refine the docking complex with the GBSW solvent model [32], CHARMm Polar H force field, and dielectric constant of 4.0. RDock energy (E_RDock) was used to evaluate the docking results [25]. The smaller the E_RDock of the docking complex, the higher the match between the receptor and the ligand.

### 3.3. MD Simulations

MD simulations were performed to fully optimize the conformation of the complex. The MD trace was also used to calculate the binding free energy using the molecular mechanics/Poisson-Boltzmann solvent accessible surface area (MM-PBSA) method [33,34] to select the dominant conformation for further analysis of the virtual mutations. The MD simulation was performed while using the Gromacs 5.1.2 software package [35,36]. The calculation system was built by using the pdb2gmx tool in the Gromacs package. The initial model was first dissolved in a cube box (7.2 × 11 × 7.2 nm cuboid box) containing 16318 SPC/E water molecules. The system was then assigned to the Amber-99sb force field [14]. The genion tool in Gromacs was used to customize the concentration of neutralized ions with the parameter setting by “-neutral-conc 0.15” to simulate the salt ion environment under physiological conditions (NaCl 0.15M) and to maintain electrical neutrality for the whole system. Finally, 52 Na ions and 60 Cl ions were added to the Box by replacing 112 water molecules.

To eliminate the conflict of atomic positions, unreasonable structural errors, including bond length and bond angle, especially the conflict between the position of water molecules, ions, and protein complexes, the simulation system was firstly minimized by performing 500-step steepest descent (SD) minimization, followed by a 2 ns position-restricted MD simulation with NVT and NPT [11] ensemble separately. Through the position-restricted MD simulation, the solvent molecules in the box can be fully dissolved with the protein complex molecule. Finally, a 600 ns production MD simulation was performed at a temperature of 310K (V-rescale thermostat), pressure at the atmospheric NPT ensemble (Parrinello-Rahman barostat), and periodic boundary conditions using Verlet cut-off scheme. The LINCS algorithm limited all of the bond lengths [22]. The electrostatic interactions were calculated using the Particle Mesh Ewald (PME) summation scheme [37]. The MD trajectories were saved every 600 ps with time step of 2.0 fs. Each MD trajectory file contained 1000 conformations.

In the MD simulations, we used the total energy and the RMSD of the backbone to examine whether the system reaches its equilibrium, and applied the cluster analysis to obtain the representative conformation after the system achieving equilibrium. All of the Gromacs MD simulation jobs were performed on the high performance computing platform of Jinan University.

### 3.4. Binding Energy Calculation by MM-PBSA

A total of 100 conformations were extracted from the equilibrium phase of each of MD including wild type and mutants. The g_mmpbsa software [38] was executed to calculate the binding free energy and residue decomposition of Ab42 and pCFH complexes using the MM-PBSA method [16,39]. In MM-PBSA, the enthalpy of the system was calculated while using the molecular mechanics (MM) method. Solving the Poisson-Boltzmann (PB) equation and calculating the molecular surface area (SA) determined the contribution of the polar part and non-polar part of the solvent effect to the free energy. The basic principle is shown in a formula, as follows:

Δ*G*_bind_ = Δ*E*_MM_ + ΔΔ*G*_sol_ − *T*Δ*S*

=Δ*E*_MM_ + Δ*G*_GB_ + Δ*G*_SA_ − *T*Δ*S*

=Δ*E*_vdw_ + Δ*E_ele_*+ Δ*G*_GB_ + Δ*G*_S_ − *T*Δ*S*,

where Δ*G*_bind_ is the binding free energy; Δ*E*_MM_ is the difference in intramolecular energy under vacuum; ΔΔ*G*_sol_ is the solvation free energy difference; *T* is the absolute temperature; and, Δ*S* is the entropy change. The Δ*E*_MM_ can be calculated by the MM method and ΔΔ*G*_sol_ is composed of polar solvation free energy difference and non-polar solvation free energy difference. While the polar part was obtained by solving the finite difference PB equation, estimating the solvent accessible SA fit the non-polar part; finally, the *T*Δ*S* was calculated while using the normal mode method.

### 3.5. CAS

Alanine scanning is to substitute an amino acid residue with alanine in the targeted region of protein, thereby replacing any functional groups on its side chain with a small neutral methyl group that exerts little effect on the protein structure [17]. In this study, the importance of key amino acids was determined by alanine scanning to calculate the changes in binding free energy before and after the amino acid substitution in order to find the key amino acid residues, which is important for the interaction between antibody Ab42 and pCFH. The CAS was performed with the Calculate Mutation Energy (Binding) module under the DS45 platform. The amino acids on the pCFH were substituted one by one with alanine. The energy differences between the wild type and the mutants in the antigen-antibody complex were subsequently determined. 

The GBSW solvent model [32,40] was used to detect the effect of the solvent, and the electrostatic terms were approximated by the sum of coulombic interactions and polar contributions to solvation energy. The van der Waals interaction energy, side-chain entropy term, and non-polar surface dependent term were also included in the energy function of the GBSW model.

### 3.6. EAS

Each amino acid residue of pCFH was substituted one by one with alanine. A total of 16 polypeptides with biotin tag, including one wild-type pCFH and 15 mutant peptides, were synthesized with a purity of 99% or more for further examination (Table 3). The ability of antibody Ab42 to bind the individual CFH mutant peptides and the CFH wild-type peptide was determined by ELISA using the method, as described [1]. Briefly, the ELISA plates were coated with 20 μg/mL streptavidin in coating solution (100 μL/well) and incubated overnight at 4 °C, washed with PBST (PBS containing 0.1% Tween 20), and then blocked with PBS containing 5% goat serum at 37 °C for two hours. After washing with PBST, 100 μL of each polypeptide at 20 μg/mL were added and incubated at 37 °C for one hour. After washing with PBST, the Ab42 antibody was added and incubated at 37 °C for one hour, and the secondary antibody, horseradish peroxidase-labeled goat anti-human IgG (H + L) antibody, was added. After incubation for another 30 min, 100 μL of TMB color developer solution was added, and the OD values were read at the wavelength of 450 nm.

## 4. Conclusions

Understanding the details of antigen-antibody interactions and identifying the important amino acid sites (epitopes) through which an antibody interacts with an antigen are critical for the development of antibody drug. The present study has applied computational simulation methods, including homology modeling, molecular docking, MD simulation, and CAS, to study the interaction between Ab42 and pCFH to explore the mechanism of interaction between human anti-CFH antibody Ab42 and its target pCFH.

The amino acid energy contributions that were obtained from both the MM-PBSA calculation and the CAS were used in our study to elucidate the key amino acids of the antigen-antibody interaction. Our results indicated that the mechanisms of interaction between Ab42 and pCFH are mainly through hydrogen bond formation and hydrophobic interaction between the amino acids Tyr315, Ser100, Gly33, and Tyr53 on the CDR of Ab42 and the amino acids Pro441, Ile442, Asp443, Asn444, Ile447, and Thr448 on the pCFH. Finally, the results that were obtained through all of the computational results were verified by the EAS method. As a native human antibody, Ab42 should have no or minimal immunogenicity in humans, and thus have better potential for development as therapeutic anti-tumor drugs without much of the concern invoking immune rejections.

In summary, the present study has important guiding significance not only for the development of Ab42 into anti-tumor drugs, but also for the investigation of other relevant antibody-antigen interactions.

## Figures and Tables

**Table 1:** The hydrogen bonds formed in the docking complex.

Donor	Acceptor
Ab42-A:Gly33:HN	Pep-C:Ile447:O
Ab42-A:Tyr53:HH	Pep-C:Gly445:O
Ab42-A:Tyr53:HH	Pep-C:Gly448:O
Ab42-B:Tyr315:HH	Pep-C:Pro439:O
Pep-C:Ile442:HN	Ab42-B:Tyr315:O
Pep-C:Asn444:HD21	Ab42-A:Ala103:O
Pep-C:Asn444:HD22	Ab42-A:Ser100:O
Pep-C:Ser449:HN	Ab42-A:Leu31:O
Ab42-A:Gly33:HA2	Pep-C:Ile447:O
Ab42-A:Ser52:HB1	Pep-C:Asp446:O
Ab42-A:Ser52:HB2	Pep-C:Asp446:O
Ab42-A:Ala101:HA	Pep-C:Asn444:OD1
Pep-C:Pro441:HA	Ab42-B:Tyr315:O
Pep-C:Thr448:HA	Ab42-A:Leu31:O
Pep-C:Thr448:HB	Ab42-A:Leu31:O
Pep-C:Pro441:HD2	Ab42-B:Tyr315:OH

**Table 2:** Complementarity-determining region (CDR) of Ab42.

CDR	a.a Position	Length (No. of a.a)	Sequence
HCDR1	26–33	8	GFTFSLYG
HCDR2	51–58	8	ISYDEKTK
HCDR3	97–107	12	AKGSAEAATLDY
LCDR1	244–255	12	QSLLYRSNKKNY
LCDR2	273–275	3	WAS
LCDR3	312–320	9	QQYYATPLT

**Table 3:** Amino acid sequence of complement factor H (CFH) wild-type and mutant peptides.

Peptide ID	Mutation Position	Sequence
WT	N/A	GPPPPIDNGDITSFP
M1	P437	APPPPIDNGDITSFP
M2	P438	GAPPPIDNGDITSFP
M3	P439	GPAPPIDNGDITSFP
M4	P440	GPPAPIDNGDITSFP
M5	P441	GPPPAIDNGDITSFP
M6	P442	GPPPPADNGDITSFP
M7	P443	GPPPPIANGDITSFP
M8	P444	GPPPPIDAGDITSFP
M9	P445	GPPPPIDNADITSFP
M10	P446	GPPPPIDNGAITSFP
M11	P447	GPPPPIDNGDATSFP
M12	P448	GPPPPIDNGDIASFP
M13	P449	GPPPPIDNGDITAFP
M14	P450	GPPPPIDNGDITSAP
M15	P451	GPPPPIDNGDITSFA
